# The effects of magnetic treatment on nitrogen absorption and distribution in seedlings of *Populus* × *euramericana* ‘Neva’ under NaCl stress

**DOI:** 10.1038/s41598-019-45719-6

**Published:** 2019-07-11

**Authors:** Xiumei Liu, Hong Zhu, Lu Wang, Sisheng Bi, Zhihao Zhang, Shiyuan Meng, Ying Zhang, Huatian Wang, Chengdong Song, Fengyun Ma

**Affiliations:** 1 Key Laboratory of State Forestry Administration for Silviculture of the lower Yellow River, Shandong Agricultural University, Taian, 271018 Shandong China; 2 Forestry College of Shandong Agricultural University, Taian, 271018 Shandong China; 3 Yichun Research Institute of Forestry Science, Yichun, 153000 Heilongjiang China; 4 Taishan Research Institute of Forestry Science, Taian, 271000 Shandong China

**Keywords:** Plant physiology, Plant stress responses

## Abstract

A potted experiment with *Populus* × *euramericana* ‘Neva’ was carried out to assess whether there are positive effects of magnetic treatment of saline water (MTSW) on nitrogen metabolism under controlled conditions in a greenhouse. Growth properties, nitrogen contents, enzyme activities and metabolite concentrations were determined based on field experiments and laboratory analysis after a 30-day treatment. The results were as follows: (1) Biomass accumulation, root morphological properties and total nitrogen content were improved by MTSW. (2) Magnetization led to a greater increase in nitrate-nitrogen (NO_3_^−^-N) content in roots than in leaves, accompanied by greater NO_3_^−^ efflux and activated nitrate reductase. (3) MTSW led to a higher ammonium-nitrogen (NH_4_^+^-N) content and greater uptake of net NH_4_^+^ in the leaves than that in the roots. (4) Magnetization stimulated glutamine synthase, glutamate dehydrogenase and glutamate synthase activities, whereas the concentrations of glutathione and oxidized glutathione were increased in leaves but decreased in roots, and the total glutathione content was increased. Overall, these results indicated some beneficial impacts of MTSW on nitrogen translocation under field conditions, especially for equilibrating the distribution of NO_3_^−^-N and NH_4_^+^-N. Moreover, these findings confirmed the potential of using low-quality water for agriculture.

## Introduction

Water scarcity is a serious problem for agricultural production in the saline areas of many countries and regions. Developing shallow brackish water resources is very costly in saline areas where fresh water is lacking^1^. As an additional water supply, brackish water is potentially a valuable source to meet crop water requirements. The development and utilization of saline water is gaining importance in agriculture in many countries, including India, China, Egypt, Russia, Ukraine, and Australia, due to issues with water quality. Although there are differences in the frequency and timing of saline water, excess soil salinity modifies the soil structure and nutrient dynamics in ecosystems with chronic salinity uptake^2^. Hence, safe and efficient use of saline water is needed for agricultural irrigation, and appropriate methods and technologies should be implemented to alleviate the excessive accumulation of soil salinity for crop production^3^. Moreover, modern efforts in agriculture and forestry are now aimed at identifying ecofriendly production technology to promote crop output.

One such potential strategy for water treatment is a magnetized technique that has been performed for centuries^4–6^, and there is potential for magnetic treatment of water to save water supplies and address the future water deficit. With well-founded scientific experiments, some studies have reported that there are beneficial effects of magnetized water irrigation on the growth, root function and chemical composition of plants^4,5,7,8^, as well as on the nutrient availability in the soil^9^. Magnetizers were employed for water treatment, and various crop responses were observed. For example, when irrigating with magnetically treated water, the seed, straw and biological yield of chickpea (*Cicer arietinum* L. var. Sena-1) increased by 39.64–41.03%^10^; additionally, in wheat, chemical components such as photosynthetic pigments, total phenols, total indole and the total number of protein bands were enhanced^11^ upon magnetic treatment relative to treatment with tap water. El-Yazied *et al*. reported that magnetically treated water irrigation led to an improvement in the phosphorus (P) content in tomato (*Lycopersicon esculentum* Mill cv. Castlrock) and in the soil, resulting in taller and heavier plants^12^. During both the vegetative and reproductive periods, magnetically treated water promoted the reducing/antioxidant capability, sugar content, protein content, etc., in lentil (*Lens culinaris* L.) relative to irrigation with common water^13^. These findings suggest the feasibility of irrigating with magnetically treated water. Moreover, some closely related studies employed a magnetized device for treatment with poor-quality water and revealed that it promoted water productivity^14^, crop production and quality, and nutrient utilization^15–17^. Several of these studies explained this evidence by showing the absorption, distribution and transportation of nutrients, such as those produced by nitrogen metabolism, in the plants in response to irrigation with water subjected to a magnetic field.

Nitrogen is an indispensable substance that contributes to the structure of proteins, nucleic acids, chlorophyll and many secondary metabolites in plants^18^. Moreover, ammonium (NH_4_^+^) and nitrate (NO_3_^−^) ions are the two main forms of inorganic nitrogen taken up by all types of plants^19^. Salinity has a strong influence on the absorption and transportation of NH_4_^+^ and NO_3_^−^ in plants. For example, fertilization with both NH_4_^+^ and NO_3_^−^ greatly alleviated the retarded growth caused by salt stress in *Catharanthus roseus*^20^. Therefore, in a saline environment, a slight increase in net NO_3_^−^ uptake and a significant reduction in net NH_4_^+^ absorption were observed in *Populus* × *canescens*^21^. Moreover, there is some evidence that the nitrogen supply to plants demonstrates a considerable interaction with the salt tolerance of plants, which has been ascribed to differences in energy requirements and interactions between Na^+^ and/or Cl^−^ uptake and the assimilation of nitrogen^22^. Similar to NO_3_^−^ acquisition, NO_3_^−^ reduction and NH_4_^+^ assimilation in response to salt stress differ according to the species or variety of plant, its intrinsic salt resistance and the nitrogenous source^23–25^.

Many of these compatible solutes are N-containing compounds, such as amino acids and betaines, and nitrogen metabolism is of central importance for salt resistance^26,27^. However, salt stress can alter the accumulation of several kinds of nitrogen-containing compounds in plant tissues^18^. Therefore, the afforestation survival rate and growth status of plants differ depending on the adaptive capacity of the plants, such as ion transport capability and compartmentalization and the synthesis and accumulation of compatible solutes. As related literature indicates the possibility that magnetic fields are a protective factor against salt stress^8^, there have been efforts to implement highly efficient utilization of saline water and minimize the toxicity caused by salinity stress through the application of magnetic techniques.

The effects of magnetically treated saline water on seedling growth and nitrogen metabolism were determined in this study under controlled conditions in a greenhouse. Furthermore, total nitrogen content, the activities of enzymes involved in nitrogen reduction and assimilation, and ion contents were studied using one-year-old *Populus* × *euramericana* ‘Neva’ (Neva hereafter) plants. Neva is widely cultivated in the Huang-Huai-Hai area of China and is reported to be sensitive to saline conditions. The main objectives of the present experiment were to examine whether there are any positive impacts on poplar growth and the metabolic capacity of nitrogen under saline stress when the plants are subjected to magnetic treatment. The findings from our experiment provide a scientific basis for the properties of magnetized water by confirming the observed changes in the physiological metabolism of plants, thus supporting exploration of the potential utilization of saline water resources in irrigation farming and other possible advantages of the use of magnetic treatment.

## Materials and Methods

### Experimental site

Field experiments were conducted at the Forestry Station of Shandong Agricultural University, Shandong Province, China (36°11′N, 117°08′E) in 2014. The region has a temperate semihumid monsoon climate, with a mean annual temperature of 13.0 °C and a mean annual global radiation of 5.09 GJ m^−2^. The mean annual precipitation is 697 mm, with precipitation concentrated from July to September. The annual average duration of sunshine is 2536.2 h, and the annual frost-free time is 195 days. The dominant soil type is brown loamy. The average bulk soil density is 1.38 g cm in the plow layer from 0–30 cm. The soil organic matter content is 24.03 g kg^−1^, and the pH is 7.8.

### Plant materials

Hardwood cuttings (12 cm in length and 1.52 cm in diameter) were taken from the middle sections of the stems of one-year-old Neva from our trial sites. In late March 2015, the cuttings were planted in ceramic pots (25 cm in diameter and 20 cm in height). The culture matrix was vermiculite. There was one cutting per pot, and there were 15 pots for each experimental treatment studied. A randomized block design was applied in the present experiment.

The plants used in the experiments were maintained in a glasshouse under natural light, with day and night temperatures of 20–25 °C, relative humidity of 60–70%, a 12-h photoperiod with 800–1000 μmol photons m^−2^ s^−1^ of photosynthetic photon flux density and irrigation with tap water. In mid-May, plants showing similar growth (20 cm in height with six leaves) were selected and cultivated in half-strength modified Hoagland solution for four weeks.

### Magnetic treatments

Before application to the seedlings, irrigation water of different salinities was treated using a magnetic device (U050 mg, 0.5 inch, output 4–6 m^3^ h^−1^) supplied by Magnetic Technologies LLC. (Russia, United Arab Emirates branch).

At the beginning of June, the plants were divided into four groups. The groups were irrigated with Hoagland solution containing 0 g L^−1^ or 4.0 g L^−1^ NaCl with or without magnetic treatment. Four groups were treated according to the methods described previously by Liu *et al*.^28^. The four groups were as follows: (1) seedlings irrigated with magnetized half-strength modified Hoagland solution containing 0 g L^−1^ NaCl (M0), (2) seedlings irrigated with half-strength modified Hoagland solution containing 0 g L^−1^ NaCl (NM0), (3) seedlings irrigated with magnetized half-strength modified Hoagland solution containing 4.0 g L^−1^ NaCl (M4), and (4) seedlings irrigated with half-strength modified Hoagland solution containing 4.0 g L^−1^ NaCl (NM4). Consequently, half of the treated pots were irrigated with magnetized brackish water, and the others were irrigated with 0 and 4.0 g L^−1^ NaCl solutions. Potted seedlings were irrigated every five days.

In the experiments, the absorption and distribution of different categories of nitrogen content as well as the ionic flux dynamics of NH_4_^+^ and NO_3_^−^, which might reflect osmotic and/or specific interactions of magnetized saline water with several steps of nitrogen metabolism, were measured based on field investigation and lab tests.

### Determination of growth characteristics of seedlings

The main characteristics of seedling growth were tested before and/or after 30-day treatment according to the methods reported by Liu *et al*.^29^. The diameters of the plants at ground level were measured using Vernier calipers, and the data were recorded. A portable leaf area meter (CI-202; CID Bio-Science, Inc., Camas, Washington, USA) was used to analyze leaf area. Leaves and roots of whole plants were collected and separated, and then the fresh weights were measured after they were rinsed in deionized water three times. The morphological parameters of roots, including the average length, the mean surface area and the total number of root tips, were determined using a WinRHIZO PRO 2007 root analysis system (Regent Instruments, Quebec, Canada) after harvest following 30-day treatment. Portions of fresh seedling samples were stored in liquid nitrogen at −80 °C. Other portions of plant samples were measured to determine dry weight after being desiccated at 105 °C, oven-dried at 80 °C, and passed through a mesh screen of 0.25 mm for analysis.

### Analysis of different categories of nitrogen

The dry samples were milled to pass through a 0.25-mm screen and achieve mixed uniformity. Fine-milled 0.5-g samples of seedling leaves and roots were digested with H_2_SO_4_-H_2_O_2_, and the concentration of total nitrogen was measured by a Kjeldahl 2300 unit (FOSS, Sweden, Germany)^30^. The nitrate-nitrogen content was determined via ethylic acid assay, and the ammonium-nitrogen content was tested by hydrogen nitrate assay following the method described by Chen^31^.

### Measurement of NH_4_^+^ and NO_3_^−^ fluxes

The net fluxes of NH_4_^+^ and NO_3_^−^ in the roots and leaves of Neva were measured at the Younger USA (Xuyue Beijing) NMT service center using noninvasive microtest technology (NMT Physiolyzer^®^, Younger USA LLC., Amherst, MA 01002, USA), along with iFluxes/imFluxes 1.0 (Younger USA LLC., Amherst, MA 01002, USA) software. Prepulled and silanized glass micropipettes (Φ5 ± 1 μm, XY-DJ-0, Younger USA) were first filled with a backfilling solution (100 mM NH_4_Cl for the NH_4_^+^ electrode; 10 mM KNO_3_ for the NO_3_^−^ electrode) for the fine roots at 10 mm and 15 mm^32^ and the mesophyll cells from tender leaves, respectively. The micropipettes were front-filled with 15–50-μm columns of selective liquid ion-exchange cocktails (NH_4_^+^ LIX, #09879, Sigma; NO_3_^−^ LIX, #72549, Sigma). A Ag/AgCl wire electrode holder (XY-DJGD, Younger USA) was inserted into the back of the electrode to make electrical contact with the electrolyte solution. YG003-Y05 (Younger USA) was used as the reference electrode. Prior to the flux measurements, the microelectrodes were calibrated for NH_4_^+^ (0.05/0.5 mM NH_4_NO_3_, 0.1 mM CaCl_2_, 0.3 mM MES, pH 5.5) with a Nernstian slope >50 mV/decade and calibrated for NO_3_^−^ (0.05/0.5 mM NH_4_NO_3_, 1 mM KCl, 0.1 mM CaCl_2_, 0.3 mM MES, pH 5.5) with a Nernstian slope <−50 mV/decade. The ionic fluxes were calculated based on Fick’s law of diffusion: J = −D_0_·(dc/dx), where J is the ion flux (unit: pmol cm^−2^ s^−1^), dc is the ionic concentration gradient, dx is the distance over which the microelectrode repeatedly moved from one point to another perpendicular to the surface of the sample at a frequency of ca. 0.3 Hz (this distance was usually between 5 and 35 μm) and D_0_ is the diffusion constant. The direction of the flux was derived from Fick’s law of diffusion.

The barehanded sections of leaves and root tips were washed three times with redistilled water. The leaves and roots were immobilized at the bottom of a chamber containing 5–10 ml of measuring buffer (for NH_4_^+^: 0.1 mM NH_4_NO_3_, 0.1 mM CaCl_2_, 0.3 mM MES, pH 5.5; for NO_3_^−^: 0.1 mM NH_4_NO_3_, 1.0 mM KCl, 0.1 mM CaCl_2_, 0.3 mM MES, pH 5.5) for 20 min and then tested by moving the ion-selective microelectrode between two positions close to the plant mesophyll cells and the elongation zone. Continuous recording was performed for 10 min at each measurement point. The steady-state ionic flux was expressed as the mean of the measured points of eight repeats; the error bars indicate the SD.

### Determination of enzyme activity and metabolite content

Nitrate reductase (NR) activity and nitrite reductase (NiR) activity were determined according to the method described by Alvarado *et al*.^33^. The samples of fresh leaves and roots were fine-ground at a ratio of 1:10 (w/v) at 0 °C by 50 mM KH_2_PO_4_ buffer with a pH value of 7.5. The homogenate was centrifuged at 3000 × g for 5 min after filtering, and the supernatant was centrifuged at 30000 × g for 20 min. The reaction mixture for NR determination contained 100 mM KH_2_PO_4_ buffer with a pH of 7.5, 100 mM KNO_3_, 10 mM cysteine, 2 mM NADH and 0.2 ml of extraction solution. The incubation was conducted at 30 °C for 30 min, and then the reaction was terminated by 0.1 ml of 1 M C_4_H_10_O_6_Zn. The reaction medium for NiR contained 50 mM KH_2_PO_4_ buffer with a pH of 7.5, 20 mM KNO_2_, 5 mM methyl viologen, 300 mM sodium dithionite and 0.2 ml of supernatant. The incubation was carried out at 30 °C for 30 min and was stopped by the oxidation of added sodium dithionite. The activities of both NR and NiR were assayed at 540 nm.

Samples of both fresh leaves and fine roots (0.5 g) were fine-ground and homogenized in 10 mM Tris-HCl buffer by a chilled mortar and pestle. The homogenates were centrifuged at 15000 × g for 30 min at 4 °C^34^. The supernatants were used to measure the activities of glutamine synthase (GS), glutamate synthase (GOGAT) and glutamate dehydrogenase (GDH). The reaction medium for GS in a final volume of 1 ml contained 80 μM Tris-HCl buffer with a pH of 8.0, 40 μM L-glutamic acid, 8 μM ATP, 24 μM MgSO_4_, and 16 μM NH_2_OH. The reaction was started by the addition of enzyme extract and stopped by 2 ml of FeCl_3_ and 5% trichloroacetic acid in 1.5 M HCl after incubation for 30 min at 30 °C. Additionally, the absorbance changes of the reaction mixture were read at 540 nm^35^. The assay medium for GOGAT, in a final volume of 3 ml, consisted of 25 M Tris-HCl buffer at a pH of 7.6 containing 0.4 ml of 20 mM L-glutamine, 0.05 ml of 0.1 M 2-oxoglutarate, 0.1 ml of 10 mmol L^−1^ KCl, 0.2 ml of 0.3 mM NADH and 0.5 ml of supernatant. The reaction began after L-glutamine was added, and the reduction in absorbance was tested spectrophotometrically at 30 °C by monitoring the oxidation of NADH at 340 nm as described by Cordovilla *et al*.^36^.

The reaction solution for the GDH test consisted of 3 ml of 0.2 M Tris-HCl buffer with a pH value of 8.0, containing 0.3 ml of 1 M 2-oxoglutarate, 0.3 ml of 1 M NH_4_Cl, 0.2 ml of 3 mM NADH and 1 ml of extraction enzyme solution. The decrease in absorbance of the 2-oxoglutarate-dependent NADH oxidation rate was recorded at 340 nm for 3 min^37^.

### Determination of GSH and GSSG

Total activation of the GSH synthetic pathway was determined by measuring GSH concentrations in the cells according to the previous method described by Baker *et al*.^38^ with some modifications^39^. The 0.5 g samples of fresh roots and leaf tissue were fine-ground and then homogenized in 0.5 ml of 5% sulfosalicylic acid with liquid nitrogen and centrifuged at 12000 × g for 10 min. Three hundred microliters of extraction solution was removed from the supernatant and 18 μl of triethanolamine at 7.5 M was added to neutralize the remaining supernatant. One hundred fifty microliters of extracted sample was used for the analysis of total glutathione (GSH + GSSG). Another 150-μl of sample was pretreated with 3 μl of 2-vinylpyridine at 20 °C for 60 min to mask GSH through derivatization, to facilitate the testing of GSSG alone. Glutathione reductase at 50 U·ml^−1^ was added into both supernatants, and the changes in absorbance were monitored at 412 nm. A standard curve was prepared and used to calculate the contents of total glutathione, reduced GSH and GSSG^40^.

### Data processing and statistical analysis

The data are reported as the mean values with standard errors (SEs) of at least three biological replicates. Statistical analysis was carried out by one-way ANOVA using SAS v. 9.0 (SAS Institute Inc., Cary, North Carolina, USA). Significant differences among four measurements were determined by Duncan’s multiple range test, with significance set at p < 0.05.

The ion flux data obtained from the ion-selective probe technique were analyzed using an Excel spreadsheet to convert data from the background -mV estimations of concentration and microvolt difference estimations of the local gradient into specific ion influxes (pmol cm^−2^ s^−1^). The microvolt differences were then exported as raw data before being imported and converted into net NH_4_^+^ and NO_3_^−^ fluxes via calculation in JCal V3.3 (a free MS Excel spreadsheet, youngerusa.com or xuyue.net)

## Results and Analysis

### Changes in seedling growth and morphological characteristics of roots

Compared with the parameters of the controls (M0, NM0), the seedling diameter, leaf area, and dry weight of the leaves and roots of Neva showed inhibition due to salt stress (M4, NM4), and all parameters showed a significant decrease (Table 1; p < 0.05), especially the biomass of leaves and roots. The biomass was reduced by 29.53% to 40.41% in leaves and by 38.79% to 48.47% in roots of plants receiving the M4 and NM4 saline treatments compared to those of the controls; the decrease in root biomass was slightly greater than that in the leaves. Compared to NM0 and NM4, the indexes of seedling growth were higher in M0 and M4 and showed a significant difference overall (p < 0.05). In particular, root biomass showed increases of 32.23% in M0 and 57.08% in M4, while the leaf biomass and area were increased by 16.94% and 20.48% in M0 and by 38.28% and 26.30% in M4, respectively, relative to values in NM0 and NM4.

**Table 1:** Growth characteristics of seedlings of Neva irrigated with magnetized and non-magnetized brackish water for 30 days.

Treatment	Diameter (cm)	Leaf area (cm^2^)	Dry weight (g)	
			Leaves	Roots
M0	0.625 ± 0.024a	55.021 ± 2.345a	3.383 ± 0.165a	0.562 ± 0.019a
NM0	0.579 ± 0.032a	45.668 ± 2.079b	2.893 ± 0.159b	0.425 ± 0.021b
M4	0.494 ± 0.027b	41.023 ± 2.844bc	2.384 ± 0.089c	0.344 ± 0.021c
NM4	0.467 ± 0.018b	32.481 ± 1.329c	1.724 ± 0.092d	0.219 ± 0.019d

Data in the table are the means ± SEs of at least three replicates. Values followed by different lowercase letters indicate significant differences at the 0.05 probability level.

For the morphological parameters of roots shown in Table 2, the results indicated that the length, surface area, volume and number of tips of roots were decreased by salt stress in the M4 and NM4 treatment groups compared with the values in the M0 and NM0 groups, and in general, the values showed remarkable variation (p < 0.05). The values were reduced by 21.55% to 66.08% in M4 relative to those in M0 and by 26.37% to 35.14% in NM4 relative to those in NM0. However, the average diameter of roots was increased by saline water, increasing by 15.52% in M4 and 15.25% in NM4 relative to the corresponding values in M0 and NM0, respectively. Compared with the parameters in NM0 and NM4, the root parameters were all increased by magnetization. In particular, the surface area, volume and number of tips of roots were increased by 222.37%, 328.88% and 165.56% in M0 and by 58.42%, 260.90% and 146.50% in M4, respectively, relative to the corresponding values in NM0 and NM4. Furthermore, the length and diameter of the roots were also increased by 13.56% to 84.50% under magnetic treatment.

**Table 2:** Morphological traits of roots of seedlings of Neva irrigated with magnetized and non-magnetized brackish water for 30 days.

Treatment	Length (cm)	Surface area (cm^2^)	Average diameter (mm)	Root volume (cm^3^)	Tips
M0	262.90 ± 14.57a	72.34 ± 4.65a	0.335 ± 0.007a	0.793 ± 0.071a	3397.33 ± 15.90a
NM0	172.35 ± 16.43ab	22.44 ± 1.59b	0.295 ± 0.005a	0.187 ± 0.049c	1279.33 ± 11.42c
M4	206.25 ± 16.54ab	24.54 ± 1.29b	0.387 ± 0.002a	0.480 ± 0.065b	2322.00 ± 11.25b
NM4	111.79 ± 10.28b	15.49 ± 1.30c	0.340 ± 0.006a	0.133 ± 0.036c	942.00 ± 10.109c

Data in the table are the means ± SEs of at least three replicates. Values followed by different lowercase letters indicate significant differences at the 0.05 probability level.

### Changes in total nitrogen

**Figure 1:**
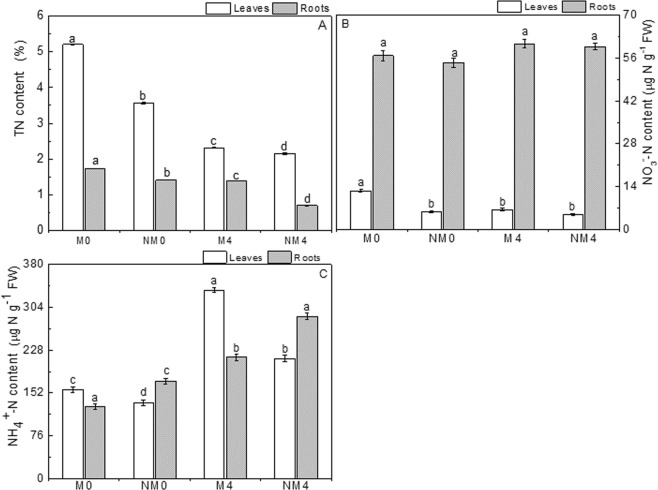
Contents of total nitrogen (TN, **A**), nitrate-nitrogen (NO_3_^−^ -N; **B**) and ammonium-nitrogen (NH_4_^+^ -N; **C**) in the leaves and roots of Neva irrigated with magnetized and non-magnetized water for 30 days. The values are the means ± SEs. Different lowercase letters show significant differences between means in the same row at the 0.05 probability level.

Irrigation with saline water led to a pronounced decrease in the content of total nitrogen in both leaves and roots, and the accumulation of total nitrogen was higher in leaves than in roots (Fig. 1A). Under salt stress, total nitrogen was reduced by 55.47% and 19.65% in M4 and by 39.61% and 50.35% in NM4 in leaves and roots, respectively, relative to the corresponding values in M0 and NM0. In contrast to the non-magnetically treated plants, the magnetically treated plants showed significant increases in total nitrogen content in leaves and roots, especially the plants in the M0 treatment group, which showed higher values than did the other three groups. Total nitrogen increased by 7.91% to 98.57%, with the maximum increase in the roots (98.57%) in M4 relative to the root total nitrogen in NM4 and an increase of 46.35% in the leaves in M0 relative to the root total nitrogen in NM0.

### Changes in NO_3_^−^-N contents

Salt stress led to decreases and increases in the NO_3_^−^-N content in leaves and roots, respectively (Fig. 1B). The NO_3_^−^-N content in roots was 7-fold greater than that in the leaves. Relative to the nitrate contents in the control, nitrate in leaves decreased by 47.79% in M4 and by 17.56% in NM4; in contrast, NO_3_^−^-N increased in fine roots by 6.89% in M4 and by 9.45% in NM4 relative to the levels in M0 and NM0, respectively. The analysis of variance revealed a significant difference between M0 and M4 (p < 0.05). For leaves, M0 showed the highest NO_3_^−^-N content, and NM4 showed the lowest content, whereas for roots, M4 had the highest content, and NM4 had the lowest content, but there were no significant differences among the four groups (p > 0.05). Generally, NO_3_^−^-N contents in leaves and fine roots were increased by magnetic treatment relative to the contents in the NM0 and NM4 treatments. There were obvious increases in the leaves of M0 and M4, with percentage increases of 115.11% and 31.74% relative to the contents in the leaves of NM0 and NM4, respectively, and M0 leaves showed the greatest increase. Although there was an increase in root NO_3_^−^-N content under magnetic treatment relative to non-magnetic treatment, the difference was not significant (p > 0.05).

### Changes in NH_4_^+^-N contents

Relative to the levels in the controls, the NH_4_^+^-N contents in leaves and roots were elevated by irrigation with saline water (Fig. 1C). There were significant differences among the four treatments (p < 0.05). The highest contents were 334.05 μg N g^−1^ FW in tender leaves of M4 and 287.58 μg N g^−1^ FW in fine roots in NM4. There were increases of 112.08% and 68.33% in M4 and 58.03% and 66.13% in NM4 in the leaves and roots, respectively, compared with the contents in M0 and NM0. The foliar content of NH_4_^+^-N showed an opposite pattern to that in roots; under magnetic field conditions, an increase in leaves was observed relative to the foliar content under non-magnetic treatment, whereas a decrease was observed in roots. The content of NH_4_^+^-N was increased by 16.99% and 57.00% in leaves and reduced by 26.20% and 25.22% in roots in the M0 and M4 treatments, respectively, relative to levels in NM0 and NM4. The differences between the magnetic and non-magnetic treatment groups were significant (p < 0.05).

### NO_3_^−^ and NH_4_^+^ ionic fluxes

#### Net fluxes of NO_3_^−^

**Figure 2:**
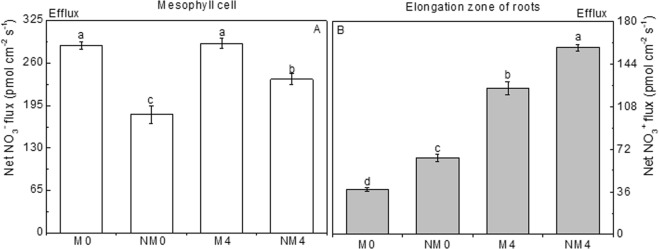
Changes in net NO_3_^−^ flux in mesophyll cells (**A**) and in the elongation region (**B**) within 10 min at 15 mm from the root tips of Neva irrigated with magnetized and non-magnetized brackish water. The means ± SEs (n > 3) of the values obtained for mesophyll cells and the elongation region are shown. Values followed by different lowercase letters represent statistically significant differences at the 0.05 probability level.

To investigate nitrate uptake in Neva after exposure to saline conditions, the NMT technique was used to monitor the net NO_3_^−^ flux in mesophyll cells and elongation zones 15 mm from the root apex, both of which showed a net efflux (Fig. 2). In the measured solution, the net NO_3_^−^ efflux increased from 235.09 to 290.23 pmol cm^−2^ s^−1^ in mesophyll cells (Fig. 2A) exposed to a NaCl solution, and there was a dramatic increase from 123.53 to 157.51 pmol cm^−2^ s^−1^ in fine roots (p < 0.05; Fig. 2B). These values were greater in plants exposed to salt stress than in the controls. NO_3_^−^ flux was increased in both leaves and roots when exposed to saline solution, and efflux was greater in mesophyll cells than in root elongation zones. Compared to the flux in the non-magnetic treatment groups, NO_3_^−^ fluxes showed remarkably higher rates in mesophyll cells exposed to magnetic field conditions. The highest net NO_3_^−^ uptake was in M4 (290.23 pmol cm^−2^ s^−1^), and the lowest uptake was in NM0 (181.42 pmol cm^−2^ s^−1^). However, the net NO_3_^−^ flux showed an opposite pattern in roots, with significantly lower efflux rates in elongation zones; the highest net NO_3_^−^ influx was in NM4 (157.51 pmol cm^−2^ s^−1^), and the lowest uptake was in M0 (38.05 pmol cm^−2^ s^−1^).

#### Net fluxes of NH_4_^+^

**Figure 3:**
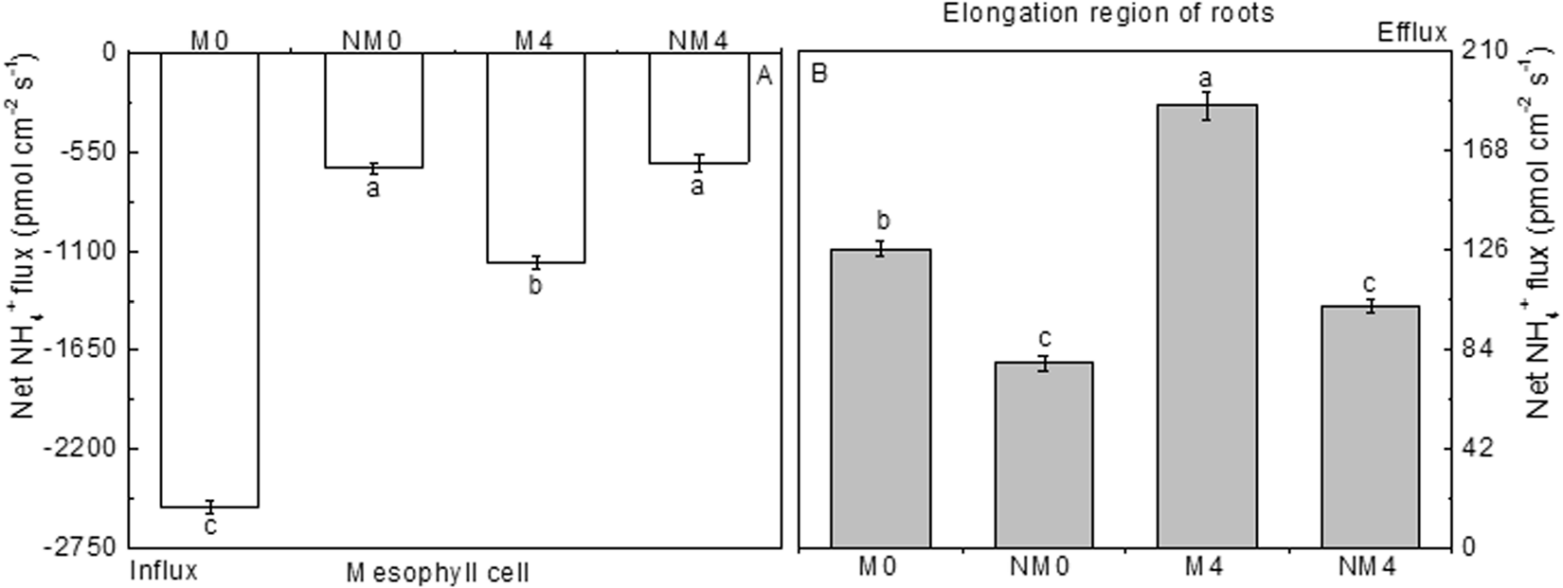
Changes in net NH_4_^+^ flux in mesophyll cells (**A**) and in the elongation region (**B**) within 10 min at 10 mm from the root tips of Neva irrigated with magnetized and non-magnetized brackish water. The means ± SEs (n > 3) of the values obtained for mesophyll cells and the elongation region are shown. Values followed by different lowercase letters represent statistically significant differences at the 0.05 probability level.

Net flux of NH_4_^+^ differed from the pattern of NO_3_^−^ uptake; there was an influx in mesophyll cells (Fig. 3A) and an efflux in fine roots (Fig. 3B). The net uptake of NH_4_^+^ in leaves showed lower absolute values in samples exposed to an NaCl solution (M4, NM4) than in samples exposed to the control treatments (M0, NM0), with M0 showing the greatest influx (−2518.85 pmol cm^−2^ s^−1^) and M4 showing the second greatest efflux (1164.15 pmol cm^−2^ s^−1^), which were significantly different (p < 0.05). Moreover, the absolute value of NH_4_^+^ flux in NM4 was very close to that in NM0, and they did not differ from each other. In contrast, the net efflux of NH_4_^+^ was higher in newly growing fine roots under salt stress than that observed in the controls, and M4 showed the highest net efflux (186.83 pmol cm^−2^ s^−1^), which was significantly different from that in M0 (p < 0.05). As was observed in leaves, the net NH_4_^+^ uptake in NM4 was similar to that in NM0 for roots, and the values showed no difference. Unlike the effects of saline treatment, the magnetic treatments induced a greater net efflux of NH_4_^+^ than did non-magnetic treatments, and both M4 and M0 showed higher values than NM4 and NM0. There was also a significant interaction between these two factors (p < 0.05).

### Activities of enzymes related to nitrate reduction and ammonium assimilation

#### NR activity

**Figure 4:**
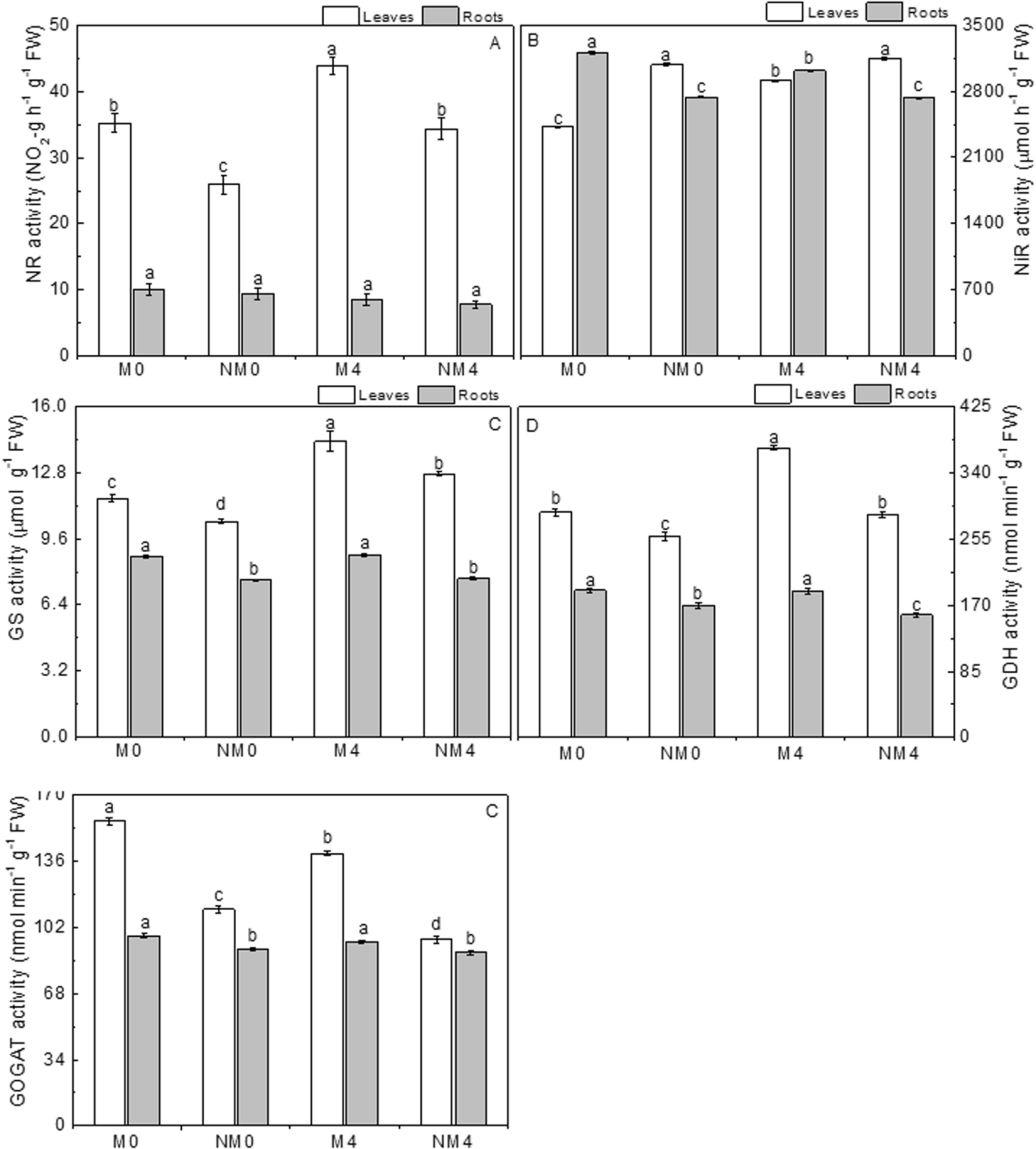
Activity of nitrate reductase (NR, NO_2_-µg hr^−1^ g^−1^ FW; **A**), nitrite reductase (NiR, μmol h^−1^ g^−1^ FW; **B**), glutamine synthase (GS, μmol g^−1^ FW; **C**), glutamate dehydrogenase (GDH, nmol·min^−1^·g^−1^ FW; **D**) and glutamate synthase (GOGAT, nmol·min^−1^·g^−1^ FW; **E**) in tender leaves and fine roots after 30 days of irrigation with magnetized and non-magnetized brackish water. The values are the means of three replicates ± SEs. Different lowercase letters show significant differences between means in the same row at the 0.05 probability level.

The NR activity of the salt-treated plants was significantly higher in the leaves than in the controls (Fig. 4A), while it was decreased in the roots. Additionally, NR activity in the leaves was higher than that in the fine roots. The salt-treated plants in the M4 and NM4 groups showed increases of 24.59% and 32.30% relative to the activities in M0 and NM0 in tender leaves, respectively. In contrast, NR activity was reduced by 15.20% and 17.12% in M4 and NM4 in fine roots compared to the activity in M0 and NM0, respectively. Unlike the inhibiting effect of non-magnetic treatments, magnetic treatments increased the NR activity in leaves and roots under saline conditions. The stimulatory effect was more obvious in the leaves than in the roots. The foliar activity of NR was significantly increased by 35.60% and 27.70% in M0 and M4 relative to the activity in NM0 and NM4 (p < 0.05), respectively. The NR activity of the roots was increased by 6.76% and 9.23% in M0 and M4 relative to the activity in NM0 and NM4, respectively. The interaction between the two factors was not significant (p > 0.05).

#### NiR activity

NiR activity showed slightly higher values under saline conditions in tender leaves than under unexposed control conditions. Moreover, saline conditions led to a decrease in the activity of NiR in the roots (Fig. 4B). Enzyme activity in leaves increased with exposure to a NaCl solution, reaching the highest value in NM4 (3145.97 μmol h^−1^ g^−1^ FW), but this was very close to the value in NM0 (3081.54 μmol h^−1^ g^−1^ FW), and the difference between the effects of the two solutions was not significant. Enzyme activity in M4 was increased by 20.25% relative to that in M0, and the difference was significant (p < 0.05). Salt treatment led to a reduction in the activity of NiR in fine roots compared to that of the controls. Enzyme activity was reduced by 0.83–6.06% in M4 and NM4 compared to the activity in the untreated controls. The highest NiR activity in roots was in M0 (3210.08 μmol h^−1^ g^−1^ FW). Under saline conditions, magnetic treatment caused a reduction in NiR activity in leaves and an enhancement of NiR activity in fine roots relative to the effects of non-magnetic treatment. NiR activity was inhibited by 7.51–21.48% in tender leaves and promoted by 10.57–17.13% in fine roots in M0 and M4 relative to the activity in NM0 and NM4, and both differences were significant (p < 0.05). The lowest activity of NiR in M0 (2419.66 μmol h^−1^ g^−1^) was in leaves, while the activity was higher in roots. The activity of NiR in NM4 was highest (3145.97 μmol h^−1^ g^−1^) in tender leaves and had a lower value (2727.77 μmol h^−1^ g^−1^) in fine roots.

#### GS activity

The enzyme activity of GS was improved to varying degrees by salt stress in both leaves and roots, and the enzyme activity in the two tissues changed in the same direction (Fig. 4C). Especially in the tender leaves, salt stress significantly enhanced GS activity by 22.03–23.79% in M4 and NM4 relative to that in M0 and NM0 (p < 0.05), but in the roots, the GS activity was not markedly different from that of the controls. Unlike the inhibitory effect on GS activity under non-magnetic conditions, the activity was obviously increased to different degrees by magnetic treatment in tender leaves and fine roots under field conditions. Enzyme activity was increased by 10.73% and 15.15% in M0 relative to that in NM0 and by 12.32% and 14.97% in M4 relative to that in NM4 for leaves and roots, respectively. The difference between the effects of magnetic and non-magnetic treatments was significant (p < 0.05). The highest GS activity was in M4, with values of 14.31 U g^−1^ FW in tender leaves and 8.83 U g^−1^ FW in fine roots. In contrast, the lowest GS activity was in NM0, with values of 10.44 U g^−1^ FW in tender leaves and 7.59 U g^−1^ FW in fine roots.

#### GDH activity

GDH activity was increased in tender leaves and decreased in roots under saline conditions compared to the activity in the controls (Fig. 4D). Salt stress stimulated the activity of GDH in leaves by 28.45% and 10.58% in M4 and NM4 relative to the activity in M0 and NM0, respectively, and the differences were significant (p < 0.05). Conversely, salt stress decreased GDH activity in fine roots by 0.71–6.92% compared to the activity in the controls. Interestingly, the enzyme activity in M4 (187.85 nmol min^−1^ g^−1^ FW) was similar to that in M0 (189.21 nmol min^−1^ g^−1^ FW). The changes in the activity of GDH showed the same trend in response to magnetic treatment compared to the effects of non-magnetic treatment seen in GS, which was enhanced both in leaves and in roots. Enzyme activity was increased by 11.87% and 11.50% in M0 relative to that in NM0 and by 29.95% and 18.92% in M4 relative to that in NM4 in leaves and roots, respectively. Moreover, the magnitude of the effect of the magnetization was slightly greater in leaves than in roots.

#### GOGAT activity

Exposure to a saline environment had an inhibitory impact on the activity of GOGAT in leaves and roots compared to the activity in the controls (Fig. 4E). The enzyme activity was markedly reduced by 10.52–14.07% in leaves and by 2.03–3.06% in roots in M4 and NM4 compared to that in M0 and NM0, respectively. Thus, the inhibitory effect of salt stress in leaves was greater than that in roots, which had values close to those of the controls. Compared to the activity under non-magnetic conditions, the activity of GOGAT was increased by varying amounts by magnetic treatment in both tender leaves and roots under field conditions. Enzyme activity showed a significant improvement in leaves (40.65–46.46%) (p < 0.05) and in roots (6.40–7.53%) compared to the activity in NM0 and NM4 due to the magnetic treatment. Additionally, the differences were significant in both leaves and roots (p < 0.05), and the stimulatory effect was greater in leaves than in roots.

#### Metabolites of nitrogen metabolism

**Figure 5:**
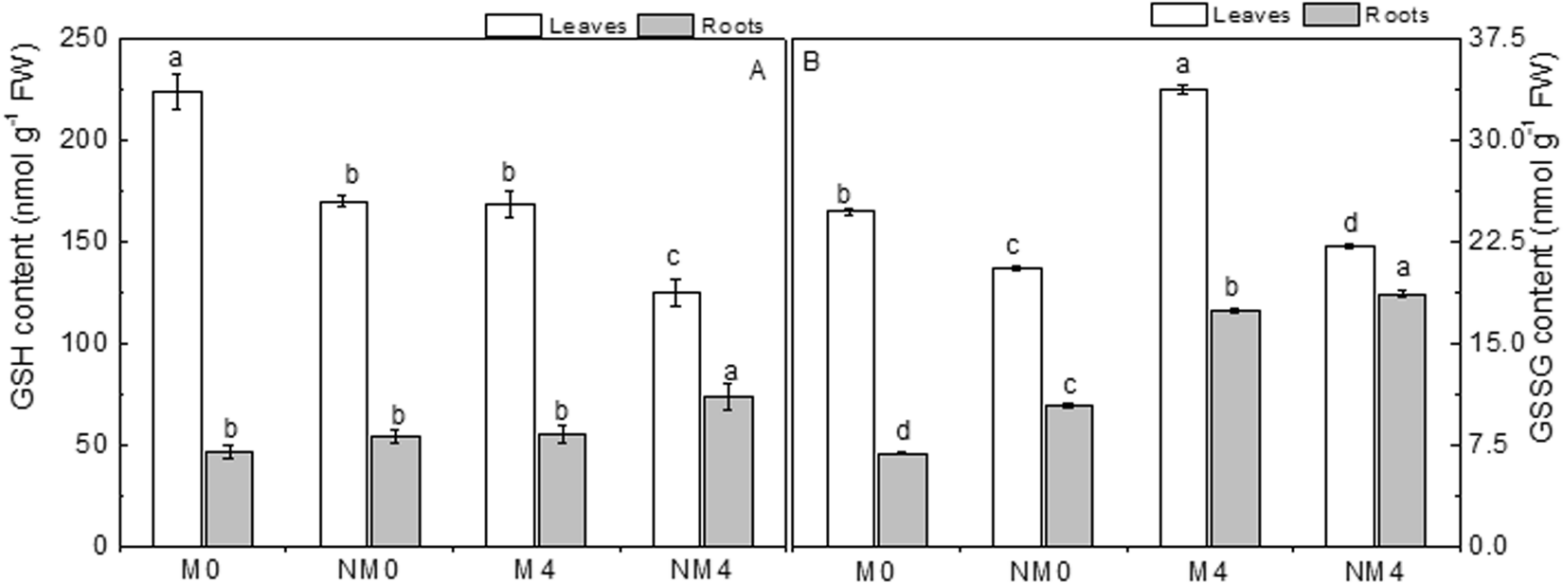
The concentrations of glutathione (GSH, μmol g^−1^ FW; **A**) and oxidized glutathione (GSSG, nmol g^−1^ FW; **B**) in tender leaves and fine roots after 30 days of irrigation with magnetized and non-magnetized brackish water. The values are the means of three replicates ± SEs. Different lowercase letters show significant differences between means in the same row at the 0.05 probability level.

Under saline conditions, the levels of GSH (Fig. 5A) were significantly decreased by 24.96–26.51% in leaves, and the levels were increased by 17.88–35.80% in the fine roots compared to the levels in unexposed controls. Magnetic treatment inhibited and stimulated the accumulation of GSH in leaves and roots, respectively. The content of GSH in leaves decreased by 32.45–34.78%, and that in roots increased by 14.09–25.29%.

The changes in GSSG content showed the same tendency in leaves and roots, with the levels showing marked increases of 7.01–153.91% under salt stress compared to the levels in the controls (Fig. 5B). The stimulatory effect of salt exposure was more obvious (78.18–153.91%) in roots than in tender leaves. Unlike the effects of salt treatment, magnetization had a stimulatory effect on GSSG levels in leaves and an inhibitory effect in fine roots. Compared to non-magnetic treatment, magnetic treatment resulted in an average reduction in leaves of 36.40% and an average increase in growing fine roots of 19.35%, and the differences between the two treatments were significant (p < 0.05).

## Discussion

### Seedling growth, ion absorption and translocation and nitrogen content of poplar

In the present study, poplar seedlings showed a reduction in biomass along with decreases in seedling diameter, leaf area, dry weight and the morphological parameters of roots (Table 1) when they were grown under NaCl stress without magnetic treatment. These findings were in accordance with the previous studies of Shrivastava and Kumar^41^ and Wani *et al*.^42^. The results of these studies showed that salt stress inhibits plant growth and development, even leading to decreases in crop output and quality; hence, most plants adapt to their environment by changing their morphological characteristics^43^. In contrast, in plants exposed to NaCl stress under magnetic field conditions, the results showed that seedling growth clearly and positively affected the measured parameters, including the dry mass of the leaves and roots. Similar results were obtained by Azita and Majd^44^, who observed that lentil seedlings grown from magnetically pretreated seeds grew taller and were heavier than untreated controls, and they showed markedly better characteristics, which is consistent with the results of our experiment. Magnetic treatment can be applied to an interface and result in the destabilization of gas bubbles, thus disturbing the ionic homeostasis between the shell of adsorbed negative ions and counter ions taken in^45^. It is likely that such interfacial effects at the water interface are responsible for the marked effects of magnetic treatment of saline water (MTSW) on Neva seedling growth in our study.

It is important to note that magnetic treatment induced an obvious acceleration in the root development of Neva seedlings (Table 2), and it is likely that a greater proportion of nutrients were allocated to the roots, thus promoting root biomass to facilitate adaptation to the saline environment^46,47^. The characteristics of root growth are of great interest in terms of the potential of roots to adjust to changes in nitrogen supply under the saline conditions of the rhizosphere, as roots are designed to explore the soil for water and nutrients^48^. The size and architecture of the root systems in our experiment indicated vigorous early growth in plants caused by magnetization, which may stimulate gene expression in the early prereplication period of plant cells exposed to magnetic fields. Furthermore, the cell reproduction cycle might be activated due to the expansion of G1 phase and G2 phase in Neva roots^49^. In addition, enhanced root growth has the potential to improve early seedling growth and thus more efficient use of nutrients by Neva. Specifically, nitrogen nutrition was improved when plants were subjected to a saline environment under magnetic field conditions through increases in the length, surface area, volume, average diameter and number of branches of roots.

Neva adjusted the saline solutions in its leaves and roots at 4.0 g L^−1^ NaCl, while the content of total nitrogen decreased, the Na^+^ content (Fig. S1C) increased, and the K^+^/Na^+^ ratio (Fig. S1A) decreased. Additionally, the Na^+^ content in roots was higher than that in leaves, whereas the total nitrogen content and K^+^/Na^+^ ratio changed in opposite directions and showed higher values in leaves than in roots. This indicates that the excessive accumulation of Na^+^ could limit nitrogen absorption and that salt recirculation from roots to leaves could reduce nitrogen concentrations. This result occurs because the water potential of the plant external environment could be lowered by the effects of Na^+^ and Cl^−^. Therefore, salinity resistance must include cellular mechanisms that contribute to osmoregulation, in part by using these ions as osmotic solutes^50^. Compared with the irrigation treatments with non-magnetized saline water, the magnetic treatments were associated with lower contents of Na^+^ in plant tissues but greater K^+^/Na^+^ ratios and total nitrogen contents (Fig. S1A), especially in the tender leaves. These findings support the trends shown for MTSW in other plants. For instance, increased K^+^ and nitrogen contents in snow pea (*Pisum sativum* L var. macrocarpon) and Kabuli chickpea (*Cicer arietinum* L.) were observed during early seedling growth^16^.

K^+^ is required by plants to maintain normal metabolic processes such as enzyme activation and protein synthesis by enabling tRNA to bind to ribosomes^51^. The ability to retain K^+^ in roots and leaves is an important mechanism of salt tolerance in poplar^52^, whereas K^+^ transport is attributed to intrinsically high plasma membrane (PM) H^+^-ATPase activity under MTSW^53^. This finding indicates that magnetic treatment has the potential to maintain internal cellular ion homeostasis. In addition, the K^+^/Na^+^ ratio can be restored either by pumping excess Na^+^ out of the cell by means of an SOS1-like^54^ and/or NhaD-like^55^ Na^+^/H^+^ antiporter at the PM level or by sequestration of Na^+^ into the vacuole of roots. Thus, magnetic treatment enhanced salt tolerance in Neva seedlings by increasing Na^+^/H^+^ antiporter activity in the tonoplast^56^, and the improved K^+^/Na^+^ ratio was beneficial for regulation at the leaf to whole-plant levels.

Nitrogen is an essential element that has been used to alleviate the toxicity caused by saline stress^57^. In our experiments, salt stress generated a decrease in NO_3_^−^-N content in tender leaves and an increase in nitrates in fine roots, with higher nitrate contents in roots than in leaves (Figs 2, 3). Interestingly, salinity led to an efflux of NO_3_^−^, causing a greater efflux in mesophyll cells than in the elongation zones of fine roots. In contrast to its effects on nitrate levels, salinity induced increases in the contents of NH_4_^+^-N in both plant tissues (Fig. 1B,C), whereas it led to a lower influx and a higher efflux of NH_4_^+^ in mesophyll cells and the elongation zones of fine roots, respectively. The results showed a reduction in nitrate uptake and an increase in ammonium uptake, which were different from the reductions in nitrogen assimilation rates for both NO_3_^−^ and NH_4_^+^ with increasing salinity in mangroves (*Kandelia candel*) found by Shiau *et al*.^58^. Moreover, the opposite changes in the ionic movement of NO_3_^−^ and NH_4_^+^ in mesophyll cells revealed that salt stress had an obvious inhibitory effect on NO_3_^−^ uptake but a stimulatory effect on NH_4_^+^ uptake, which might be attributed to the influx of NH_4_^+^ in mesophyll cells and the greater efflux of NO_3_^−^, which would enhance the uptake of ammonium in Neva. These results are inconsistent with previous findings in *Salvinia natans* and *Salvinia molesta* of a preference for NH_4_^+^ over NO_3_^−^, with higher growth rates observed in plants grown on NH_4_^+^ than in those grown on NO_3_^−^ ^59^. These patterns are potentially due to the lower amount of energy needed for NH_4_^+^ influx and the prevalence of NH_4_^+^-N in water-saturated anoxic soils^60,61^.

The contents of NO_3_^−^-N and NH_4_^+^-N and the efflux of NO_3_^−^ showed higher values in both roots and leaves in plants under MTSW than in non-magnetically treated plants. Furthermore, magnetization had a stimulatory effect on nitrogen uptake in tender leaves but a more limited effect in fine roots. Nonetheless, NO_3_^−^-N contents were slightly higher in roots than in leaves. In contrast, efflux of NO_3_^−^ was greater in mesophyll cells than in fine roots, which reflected the high net uptake of NO_3_^−^ in the fine roots of Neva. This result is consistent with a salt-enhanced NO_3_^−^ net influx observed in the root region of the mature sweet potato cultivar Xu 22 (*Ipomoea batatas* L.)^62^, suggesting that this process may contribute to normal nitrogen assimilation and that the cytosolic concentrations of NO_3_^−^ were lower than the threshold levels of nitrogen assimilation needed to support growth^32^. NO_3_^−^ uptake is required for PM-H^+^-ATPase activity^32^, and H^+^ uptake was improved in the apical areas of Neva roots^28^. These results indicated that the 2 H^+^/NO_3_^−^ symport system stimulated by magnetization could contribute to the observed NO_3_^−^ intake under salt stress. As transporters mediate NO_3_^−^ uptake across the PM in roots induced by magnetization, an essential element in the process of NO_3_^−^ assimilation is the trafficking of the NO_3_^−^ ion across membranes^63^. Poplars are fast-growing species and have high demands for nitrogen^64^; therefore, strong uptake of NO_3_^−^ and high NO_3_^−^-N contents in fine roots were expected after magnetic treatment when the plants were exposed to saline conditions.

Unlike the effects on NO_3_^−^ efflux, the magnetization triggered a net NH_4_^+^ uptake in mesophyll cells and greater efflux in growing fine roots, which caused a rapid increase in the NH_4_^+^ concentrations in plant cells under field conditions compared to the levels in the non-magnetically treated plants. In general, there were some indications that the mesophyll cells markedly preferred NH_4_^+^ to NO_3_^−^ under the influence of magnetization; assimilation of NH_4_^+^ as the fixed form of nitrogen might be attributed to the protein synthesis and lower energy expenditure induced by MTSW. Smart and Bloom *et al*.^65^ observed that high concentrations of NH_4_^+^ did not impede root growth. In contrast, we found a lower NH_4_^+^-N content and a greater efflux of NH_4_^+^ in the elongation zones of roots in association with magnetization and a lower NH_4_^+^ uptake rate in roots than in the leaves. These observations suggest that the assimilation of ammonium into amino acids generates a proton that is typically released into the rhizosphere, as stimulated by magnetization. Accordingly, ammonium utilization should acidify the environment of the root tip and accelerate root elongation because cell-wall extensibility is controlled, at least partially, by pH^66^. Hence, the lower accumulation of NH_4_^+^ could better contribute to root formation when irrigating with magnetized saline water.

### Nitrogen metabolism and translocation in leaves and roots of poplar

Nitrate is absorbed by plant roots and then converted to ammonium by the sequential reductive action of NR and NiR^67^. External salinization influences enzyme activity, causing salt-dependent regulation. The NH_4_^+^ derived from NO_3_^−^ reduction is first converted to glutamine by GS and then to glutamate by GOGAT. Subsequently, following the NR- and NiR-mediated reduction of NO_3_^−^, the absorbed NH_4_^+^ is incorporated into amino acids by GS and GOGAT (Fd-GOGAT and NADH-GOGAT) or through the alternative GDH pathway^68,69^. Debouba *et al*. found that the activity of NR in tomato plants (*Lycopersicon esculentum* Mill) was repressed in leaves but enhanced in roots under saline conditions, while NiR activity declined in both leaves and roots; additionally, GDH was inhibited by salt stress^70^. Singh *et al*. found that the enzymes responsible for assimilating NO_3_^−^ and NH_4_^+^, such as NR, NiR, GS and GOGAT, were adversely impacted by NaCl stress, while GDH exhibited the opposite trend in tomatoes (*Solanum lycopersicum* L.)^71^.

In the present study, the activity of NR was increased in tender leaves and decreased in roots, and overall, foliar NR activity was higher than root NR activity (Fig. 3A). These findings are consistent with the elevated rate of nitrate reduction in the leaves of *Zea mays* relative to the rate in its roots^72^. The enhanced activity of NR occurring in Neva leaves might be related to higher NR protein levels and sufficient availability of light and reducing power^73^, which is in agreement with the increased biomass output of leaves relative to roots in our experiment. In contrast to NR activity, NiR activity was repressed in both plant tissues in the tested Neva seedlings under NaCl stress (Fig. 3B). Furthermore, we found that the NO_3_^−^-N content was higher in roots than in leaves, which might have been due to the stimulated activity of NR and the greater net efflux of NO_3_^−^ in mesophyll cells along with the increased NiR activity in roots. This difference could result in a promotion of NO_3_^−^ flux from leaves to roots, thus leading to increased levels of NO_3_^−^ in the roots. The present results indicated that magnetization positively affected the activity of NR in both plant tissues under saline conditions. However, we observed that the activity of NiR was negatively influenced in leaves but positively affected in fine roots; in contrast, the net NO_3_^−^ flux and NO_3_^−^-N content showed different changes. Considering the patterns of NO_3_^−^ flux and NO_3_^−^-N content, we speculate that the magnetization resulted in decreases in NO_3_^−^ efflux and nitrate content followed by increases in the activities of NR and NiR in the fine roots of Neva irrigated with saline water. This finding indicated that Neva suffered from a lower concentration of NO_3_^−^-N due to MTSW.

Because low-external NO_3_^−^ supplies (iHATS) can activate some high-affinity systems (HATs) and some HATS are constitutively expressed (cHATS), there was a transient improvement in the abundance of *AtNRT2.1* mRNA. Therefore, magnetic treatment may result in transfer from NO_3_^−^ to nitrogen-free medium. However, previous studies have supplied no information on *NRT2* in leaves, which is presumably regulated by the nitrogen levels in roots^74^. For NO_3_^−^ uptake, both high- and low-affinity transport systems are necessary^75^, and how the characteristics of NR and NiR gene expression in the absence of nitrogen are affected by magnetic treatment in roots and leaves is still not clear from our studies. Otherwise, the enhanced NR activities were consistent with the changes in NH_4_^+^-N, NO_3_^−^ -N and total nitrogen contents, and all of these results confirmed that MTSW promoted the reduction and assimilation of nitrogen.

The ammonium-assimilating enzymes GS, GOGAT and GDH play significant roles in plant growth and development because the glutamate resulting from ammonium assimilation is used for the synthesis of several other amino acids (Fig. 3C–E). In the present study, NaCl had a stimulating effect on GS and an inhibitory effect on GOGAT in both plant tissues, whereas salt stress had inhibitory effects in *Solanum lycopersicum*^71^. Unlike the greatly decreased activity in the leaves and roots of *Lycopersicon esculentum*^70^, our results showed that GDH activity increased in tender leaves and decreased in fine roots. These findings illustrated that the aminating activity of GDH can be stimulated by salt stress^76^. Furthermore, GDH is required *in vivo* in ammonium detoxification and for supplementing the glutamate pool; high glutamate concentrations are required to generate protective metabolites. Generally, these conditions can perturb the activities of enzymes, such as the activation of GS and the inhibition of GOGAT. GS, GOGAT and GDH showed the same changes, with salinity causing greater activation in tender leaves than in roots, indicating impaired ammonium assimilation, which was evident from the decrease in net NH_4_^+^ uptake and increased content of NH_4_^+^-N. It seems that NaCl stress transferred nitrogen metabolism from the roots to the leaves in an attempt to sustain plant growth.

Under MTSW, high expression levels and enzymatic activities of GS, GOGAT and GDH, which are involved in the GS/GOGAT cycle, were observed in both tender leaves and roots, ensuring high glutamate concentrations. These results are consistent with the findings that GS, GSH and GOGAT activities in the leaves and roots of Chinese cabbage (*Brassica campestris* L. ssp. *chinensis* Makino cv. Aijiaohuang) were obviously enhanced by poly(γ-glutamic acid) (γ-PGA) at normal Ca^2+^ levels and that γ-PGA increased the content of total nitrogen in leaves^77^. These observations showed that magnetization stimulated the accumulation of total nitrogen at a high concentration under salt stress in Neva. The response of plants to abiotic and biotic stimuli or stress, for instance, salinity, chilling injury and drought, have been found to differ with enhanced cytoplasmic Ca^2+^ content^78,79^. However, we found that the content of Ca^2+^ (Fig. S1B), which is a necessary nutrient for seedling growth in Neva, was increased in both the leaves and roots of plants under salt stress in association with magnetic treatment. This increase caused an increase in the [Ca^2+^]_cyt_ concentration, and the activities of key enzymes in nitrogen metabolism were significantly promoted. Therefore, nitrogen metabolism was enhanced by magnetization via stimulation of the activation of Ca^2+^ as a universal second messenger in plant signal transduction, which could influence many important physiological and biochemical processes^80,81^, thereby promoting plant growth.

The present findings revealed the modulation of GSH and GSSG contents (Fig. 5) and significantly reduced the levels of total glutathione (GSH + GSSG, Fig. S2B) when Neva seedlings were subjected to saline conditions. Saline stress markedly suppressed foliar levels of GSH and stimulated GSH accumulation in the roots, and GSSG contents were increased by salt treatment in both the leaves and the roots. Moreover, we found that NaCl stress induced greater accumulation of both GSH and GSSG in the leaves than in the roots. The GSH content was reduced in response to salt stress in the seedlings, correlating with the high GSSG content, which was similar to the results observed in sodium nitroprusside-treated sunflower seedlings^82^. NaCl promoted the contents of GSH and GSSG in the leaves of poplar, which could improve the adaptive capability of this plant to saline conditions. Similarly, magnetization appeared to aid in the equilibration of synthesis, degradation, utilization and translocation (short- and long-distance) of GSH and GSSG when Neva was exposed to saline conditions. There were also higher contents of GSH and lower contents of GSSG, with higher contents in the leaves and lower contents in the roots, in plants irrigated with magnetized saline water than in non-magnetically treated plants. Recent earlier observations indicated a marked reduction in the activity of glutathione peroxidase (GPX) in cotyledons accompanying the sensing of NaCl stress. GPX activity results in the accumulation of GSSG, a substrate for GR, and a reduction in its activity results in higher GSH levels in the tissue^83^. The results in the present study showed similar changes, indicating that magnetization could modulate the distribution levels of GSH and GSSG in leaves and roots. Perhaps magnetization stimulates the pathway of glutathione synthesis by modulating the expression of the GSH gene, thereby enhancing GSH biosynthesis, especially in leaves, and leading to greater tolerance to salt stress in plants. Obviously, the different modulatory mechanisms related to GSH and GSSG affected by magnetization under saline conditions are still not clear from our studies, and more research is needed.

## Conclusions

1. The magnetic treatment of irrigation water led to an improvement in seedling growth and the morphological characteristics of roots by stimulating the uptake and assimilation of nitrogen nutrients.
2. The metabolic ability of nitrogen nutrition was promoted in the leaves and roots of poplar by a magnetic field via regulation of the enzyme activities of NR, GS and GOGAT. Moreover, the ionic dynamics of NO_3_^−^ and NH_4_^+^ in mesophyll cells and in the elongation zone of roots were affected by nitrogen translocation, which even helped to regulate the distribution of NO_3_^−^-N and NH_4_^+^-N. Moreover, the findings regarding NO_3_^−^ efflux and NH_4_^+^ influx could be valuable for investigating the preferences of Neva for NH_4_^+^ relative to NO_3_^−^, especially in tender leaves upon exposure to magnetic treatment in a saline environment.
3. Magnetized water irrigation was beneficial in maintaining the homeostasis of a variety of salt ions by reducing Na^+^ content and increasing Ca^2+^ content and the K^+^/Na^+^ ratio in poplar. Thus, it improved the ability of plants to adapt to the saline environment.
4. Overall, the data collected in this preliminary study describe a mechanism at the metabolic level under field conditions, suggesting that there may be some beneficial effects of irrigation with MTSW for plants. Additionally, the molecular mechanism underlying the response to salinity stress in poplar and the influence of soil irrigation with magnetically treated saline water need to be further studied to reveal the beneficial magnetization-mediated effects on plant growth and the soil microenvironment.

## Supplementary information

SUPPLEMENTS
