# Supplementary material for: The effects of magnetic treatment on nitrogen absorption and distribution in seedlings of Populus × euramericana ‘Neva’ under NaCl stress
Source: Sci Rep. 2019 Jul 11;9:10025. doi: 10.1038/s41598-019-45719-6 (PMC6624201; doi:10.1038/s41598-019-45719-6)
Supplement: Supplementary file 1 — SUPPLEMENTS [file 41598_2019_45719_MOESM1_ESM.docx]

**Title:**

The effects of magnetic treatment on nitrogen absorption and distribution in seedlings of *Populus × euramericana* 'Neva' under NaCl stress

**Author names and affiliations:**

Xiumei Liu^1,2^, Hong Zhu^1,2^, Lu Wang^1,3^, Sisheng Bi^1,2^, Zhihao Zhang^1,2^, Shiyuan Meng^1,2^, Ying Zhang^1,2^, Huatian Wang^1,2^, Chengdong Song^4^, Fengyun Ma^1,2^*

^1^Key Laboratory of State Forestry Administration for Silviculture of the lower Yellow River, Shandong Agricultural University, Taian 271018, Shandong, China; ^2^Forestry College of Shandong Agricultural University, Taian 271018, Shandong, China; ^3^Yichun Research Institute of Forestry Science, Yichun 153000, Heilongjiang, China; ^4^Taishan Research Institute of Forestry Science, Taian 271000, Shandong, China

Figure S1 Ratio of potassium to sodium (K^+^/Na^+^, A), calcium content (Ca^2+^, B) and sodium content (Na^+^, C) in leaves and roots of Neva irrigated with magnetized and non-magnetized water for 30 days. The values are the means of three replicates ± SEs. Different lowercase letters indicate significant differences between means in the same row at the 0.05 probability level.

Figure S2 Total contents of glutathione (GSH, μmol g^-1^ FW) and oxidized glutathione (GSSG, nmol g^-1^ FW) in tender leaves and fine roots; (A) total glutathione content (the total contents of GSH and GSSG, B) after a 30-day irrigation with magnetized and non-magnetized brackish water. The values are the means of three replicates ± SEs. Different lowercase letters indicate significant differences between means in the same row at the 0.05 probability level.
